# Carbon Inks-Based Screen-Printed Electrodes for Qualitative Analysis of Amino Acids

**DOI:** 10.3390/ijms24021129

**Published:** 2023-01-06

**Authors:** Teodor Adrian Enache, Monica Enculescu, Mihaela-Cristina Bunea, Estibaliz Armendariz Zubillaga, Edurne Tellechea, Maite Aresti, María Lasheras, Aaron C. Asensio, Victor C. Diculescu

**Affiliations:** 1 Laboratory of Functional Nanostructures, National Institute of Materials Physics, Atomistilor 405A, 077125 Magurele, Romania; 2 NAITEC, C/Tajonar 20, 31006 Pamplona, Spain; 3 Mateprincs S.L., Centro Stirling, Carretera de Tafalla 10, 31132 Villatuerta, Spain

**Keywords:** screen-printed electrodes, modified carbon ink, PANI/ZnO nanowires, quantitative analysis of amino acids, tyrosine, tryptophan

## Abstract

Due to the great significance of amino acids, a substantial number of research studies has been directed toward the development of effective and reliable platforms for their evaluation, detection, and identification. In order to support these studies, a new electrochemical platform based on PANI/ZnO nanowires’ modified carbon inks screen-printed electrodes was developed for qualitative analysis of electroactive amino acids, with emphasis on tyrosine (Tyr) and tryptophan (Trp). A comparative investigation of the carbon ink before and after modification with the PANI/ZnO was performed by scanning electron microscopy and by Raman spectroscopy, confirming the presence of PANI and ZnO nanowires. Electrochemical investigations by cyclic voltammetry and electrochemical impedance spectroscopy have shown a higher charge-transfer rate constant, which is reflected into lower charge-transfer resistance and higher capacitance values for the PANI/ZnO modified ink when compared to the simple carbon screen-printed electrode. In order to demonstrate the electrochemical performances of the PANI/ZnO nanowires’ modified carbon inks screen-printed electrodes for amino acids analysis, differential pulse voltammograms were obtained in individual and mixed solutions of electroactive amino acids. It has been shown that the PANI/ZnO nanowires’ modified carbon inks screen-printed electrodes allowed for tyrosine and tryptophan a peak separation of more than 100 mV, enabling their screening and identification in mixed solutions, which is essential for the electrochemical analysis of proteins within the proteomics research field.

## 1. Introduction

The starting point of protein electrochemistry dates back to the 1970s and 1980s when several studies demonstrated that tyrosine- (Tyr) [1,2], tryptophan- (Trp) [1,2], histidine- (His) [1,2], and sulfur-containing amino acids, namely cysteine (Cys) [1,2,3,4] and methionine (Met) [1,4], are oxidizable at gold, platinum, or carbon electrodes. Until the end of 1980s, it was already demonstrated that the redox properties of amino acids, especially Tyr and Trp, allow the investigation of proteins [1,2]. The last decade came with the elucidation of electroactive amino acids oxidation mechanisms [5,6,7,8,9], while the research was extended to broad studies regarding different phenomena such as adsorption [9], aggregation [10], fibrilization [11], structural modifications [12], protein/enzyme–substrate interaction [13], etc., and the investigation addressed to peptides [14], amyloids [15,16], proteins [15,17], and enzymes [14]. At the same time, in the last decade, this field of electrochemistry has been governed by the use of glassy carbon working electrodes [18] and other carbon electrodes, such as disposable carbon-based screen-printed electrodes [19], mainly due to their broad potential window, which is usual between −1.0 V and +1.4 V [18,20,21,22]. Due to its extensive use, the glassy carbon electrode is generally accepted as a standard regarding the oxidation potential of amino acids, as shown in Table 1.

As can be seen in Table 1, the oxidation of amino acids at glassy carbon electrode, at pH = 7.0, occurs in one (Tyr and His), two (Trp and Met), or three (Cys) steps. Considering their current/concentration ratio, for general application, tyrosine and first tryptophan oxidation reactions present the highest interest. Comparing with other electroactive amino acids at the same concentration, tyrosine and tryptophan oxidations at glassy carbon electrodes take place with the highest currents, a fact that recommends these amino acids for analytical proposals, especially in a complex matrix.

Given that the oxidation of tyrosine and tryptophan at the glassy carbon electrode takes place at the same potential, for the field of protein electroanalysis, it is necessary to use a different electrode material, which allows the separation of their oxidation potentials while maintaining a high current/concentration ratio. This problem was partially solved by using a boron-doped diamond electrode [9], but improvement is still needed.

On the other hand, the use of polymers and nanomaterials for the fabrication of sensors and biosensors provides solutions to common inconveniences such as the overlapping of the amino acids redox potentials [23]. In the last five years, numerous research groups in the field of electroanalysis as well as in the field of materials science have developed modified electrodes with conducting polymers to be employed as sensors and biosensors for a wide range of analytes [24,25]. Numerous studies were carried out regarding polypyrrole (PPy) [26,27], polyaniline (PANI) [28,29], and poly(3,4-ethylenedioxythiophene) (PEDOT) [30], etc. PANI is often a choice in electrochemical sensor fabrications, as it is used as the matrix for nanocomposites employed to modify screen-printed electrodes for various electrochemical sensing studies [31,32,33]. Furthermore, zinc oxide nanocomposites are often used to modify the screen-printed electrodes in order to fabricate fast and efficient non-enzymatic electrochemical sensing platforms for detecting amino acids [34,35,36].

In order to discriminate the oxidation potentials of tyrosine and tryptophan, an essential process in the field of electrochemistry, the aim of this work was to develop and fabricate new conductive materials to be used as electrodes able to discriminate the oxidation reactions of tyrosine and tryptophan. The goal was not only to achieve this performance but also to provide a low-cost electrode that is easy to use and ready for mass production. This was accomplished through the screen-printing of a planar three-electrode system consisting of PANI/ZnO-functionalized carbon ink as working electrode, carbon as auxiliary electrode, and silver and pseudo-reference electrode fabricated on the same flexible, polyethylene terephthalate surface.

## 2. Results and Discussion

A planar three-electrodes system consisting of a carbon-based working electrode, a silver counter electrode, and a silver reference was fabricated on a PET substrate using the screen-printed method. This electrode system was mass-produced and hereafter will be called SPE (screen-printed electrode). By modifying the working electrode ink with nanostructured materials, i.e., PANI/ZnO nanowires’ composite, a modified planar three-electrodes system (mSPE) was fabricated. The mSPEs were produced in a stepwise procedure, with different PANI/ZnO proportions, as shown in Table S1. The analysis/screening of their electrochemical response was performed by cyclic voltammetry in different electrolytes such as 0.1 M H_2_SO_4_, 0.1 M HCl, and pH = 7.0 0.1 M phosphate buffer, in the absence and in the presence of 1 mM K_4_[Fe(CN)_6_], as shown in Figure S1. The electrode with 2% (*w*/*w*) PANI and 0.04% (*w*/*w*) ZnO nanowires showed the most appropriate electrochemical response and was chosen for further investigations.

### 2.1. Morphological Characterization of Carbon-Based Screen-Printed Electrodes

**Figure 1:**
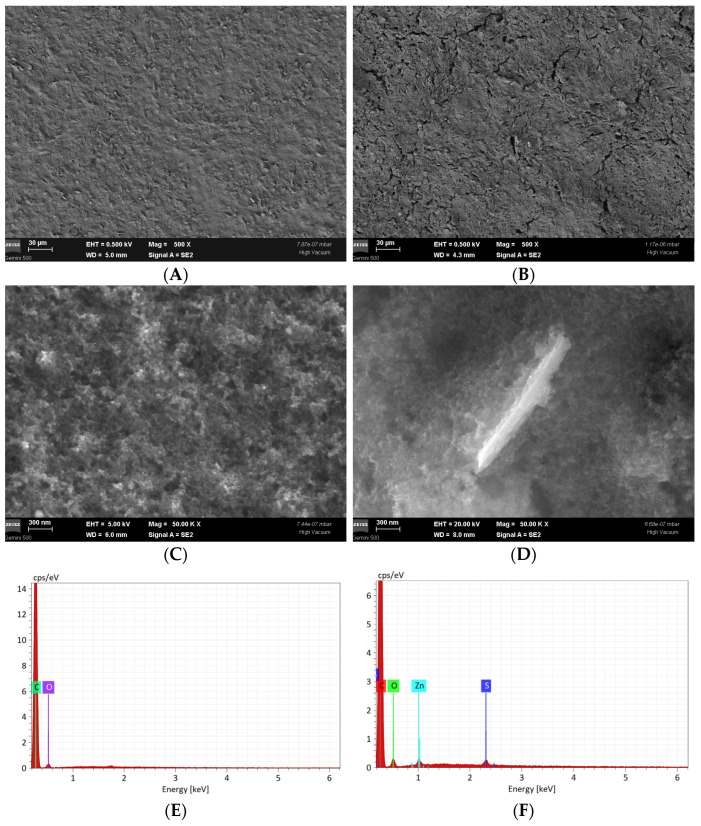
FESEM images of working electrode’s surface at different magnifications (500× and 50,000×) for SPE (**A**,**C**) and mSPE (**B**,**D**) and EDX spectra for SPE (**E**) and mSPE (**F**).

Morphological characterization of the screen-printed three-electrode systems (SPE and mSPE) was performed using field emission scanning electron microscopy (FESEM) (Figure 1) in order to highlight the modifications of the surface after the use of nanostructured PANI/ZnO nanowires in the composition of carbon-based inks.

Figure 1A presents the surface of the SPE. The image of the surface of the mSPE, modified using the PANI/ZnO composite, as shown in Figure 1B, revealed the increase of the roughness of the surface of the electrode but while preserving the uniformity of carbon based ink, which is essential in order to maintain a comparable morphology of the screen-printed electrode.

The changes in the morphology produced by the modification of SPE with the PANI/ZnO nanowire composite are better observed in the higher-resolution images. Thus, while only the nanostructuring produced by the carbon-based ink is observed in the FESEM image of SPE (Figure 1C) the image of the PANI ZnO-modified electrode system, mSPE, demonstrates the incorporation of the ZnO nanowires in the mSPE’s surface (Figure 1D). The presence of ZnO in the composition of the modified ink was confirmed by EDX measurements where the spectrum of SPE revealed only the presence of C and O (Figure 1E), while Zn was observed in the elemental composition of the mSPE (Figure 1F).

**Figure 2:**
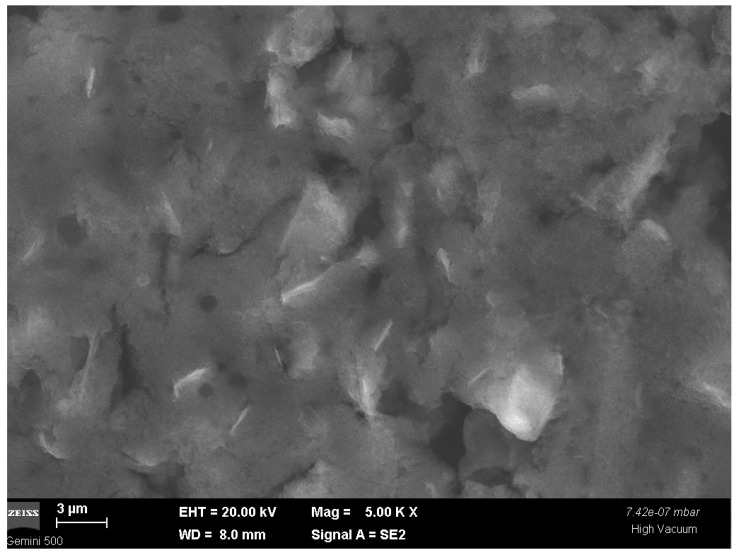
FESEM image of mSPE surface demonstrating the presence of ZnO nanowires in the modified carbon-based ink.

The number of ZnO nanowires incorporated within the PANI/ZnO-modified ink is proportional with the ZnO nanowires’ concentration in the carbon-based ink. At a magnification of 5000×, a number of nanowires can be observed on the surface of mSPE, thus increasing the roughness of the mSPE surface and also enhancing the surface area of the working electrode, as shown in Figure 2.

### 2.2. Structural Characterization by Raman Spectroscopy

**Figure 3:**
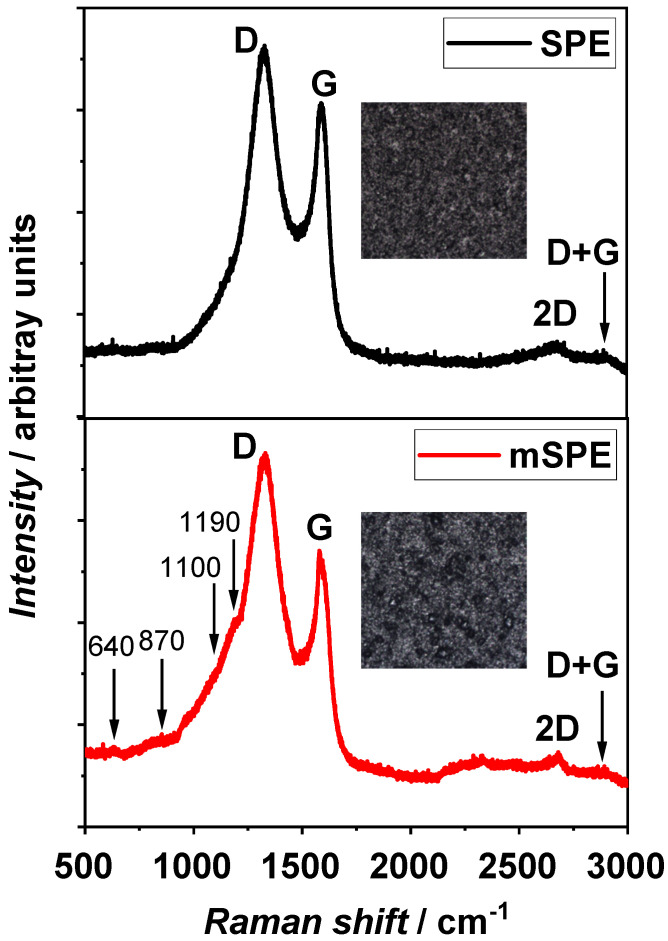
Raman spectra of SPE and mSPE for 633 nm excitation. Insets: optical images of evaluated electrodes’ surfaces.

Raman spectroscopy was employed to characterize the surface of the carbon ink screen-printed electrodes, as shown in Figure 3. The features characteristic to carbon ink electrodes can be observed in both reference and modified electrodes.

The main features in the Raman spectra of carbon ink-based electrodes are two characteristic peaks in the 1300–1600 cm^−1^ domain, namely the D and G bands, respectively (Figure 3). The assignment of the D and G peaks is straightforward for carbonous materials. The intense peak appearing at 1330 cm^−1^ called the D band is related to the presence of defects in the carbonous material, and the G band that appears at 1588 cm^−1^ is associated to in-plane vibration modes of sp^2^ hybridized carbon atoms [37,38,39]. Additional characteristic features can be observed at higher wavenumbers: a third band so-called the 2D band peaking at 2663 cm^−1^ that is due to the second-order Raman scattering of the D band and a weak defect-activated Raman peak around 2903 cm^−1^ that is known as the D+G band and is associated with combination scattering [40,41].

Besides the carbon ink-related bands, the Raman spectra of PANI/ZnO-modified electrode reveals additional features induced by the PANI/ZnO composite. Although the bands induced by the presence of PANI and ZnO show very low intensities due to the low concentration of the PANI and ZnO nanowires in the carbon ink, the contributions of ZnO are still visible at about 640 cm^−1^ and 870 cm^−1^ [42,43], shifted to shorter wavenumbers due to the interparticle interactions. In the Raman spectra of the mSPE, one can observe the PANI-induced broadening of the bands in the 1100–1600 cm^−1^ region where the main Raman features of PANI are present, with two distinct shoulders appearing at 1100 cm^−1^ and 1195 cm^−1^ [42,43]. In addition, the large contribution in the 2000–2500 cm^−1^ domain is induced by the PANI/ZnO composite.

The ratio between the intensities of the D and G peaks can be also an indication of the presence of defects in the carbon material disorder induced by the PANI/ZnO composite. An increase of the I_D_/I_G_ ratio from the value of 1.17 for the reference electrode to the value of 1.35 for the modified electrode indicates that a larger number of defects are present on the surface of the PANI/ZnO-modified carbon ink electrode, as expected.

### 2.3. Electrochemical Characterization of Carbon-Based Screen-Printed Electrodes

The electrochemical characterization of the SPE and mSPE electrodes was initially carried out by cyclic voltammetry in different electrolytes such as 0.1 M H_2_SO_4_, 0.1 M HCl, and 0.1 M phosphate buffer pH 7.4 (Figures S1 and S2). While SPE showed a large potential window comprising the interval between −0.20 V and +1.20 V without the occurrence of charge-transfer reactions, the PANI:ZnO-modified electrodes showed peaks corresponding to the PANI redox process. The stepwise increase of the ZnO ratio within the PANI:ZnO composite led to the decrease of the PANI redox peaks, as observed on the voltammograms recorded in acid media. Another observation is that the increase of the ZnO ratio within the PANI:ZnO composite led to an increase in the PANI conduction properties in neutral media, as observed on CVs recorded in pH = 7.0 0.1 M phosphate buffer. These experiments demonstrated that ZnO acts as a dopant of PANI, increasing its conductivity. Nonetheless, as already mentioned, the electrode with 2% (*w*/*w*) PANI and 0.04% (*w*/*w*) ZnO nanowires (further denominated as mSPE) showed the most appropriate electrochemical response and was chosen for further investigations.

A thorough investigation of SPE and mSPE was also performed in the presence of 1 mM K_4_[Fe(CN)_6_] as a redox probe in the pH = 7.0 0.1 M phosphate buffer supporting the electrolyte.

**Figure 4:**
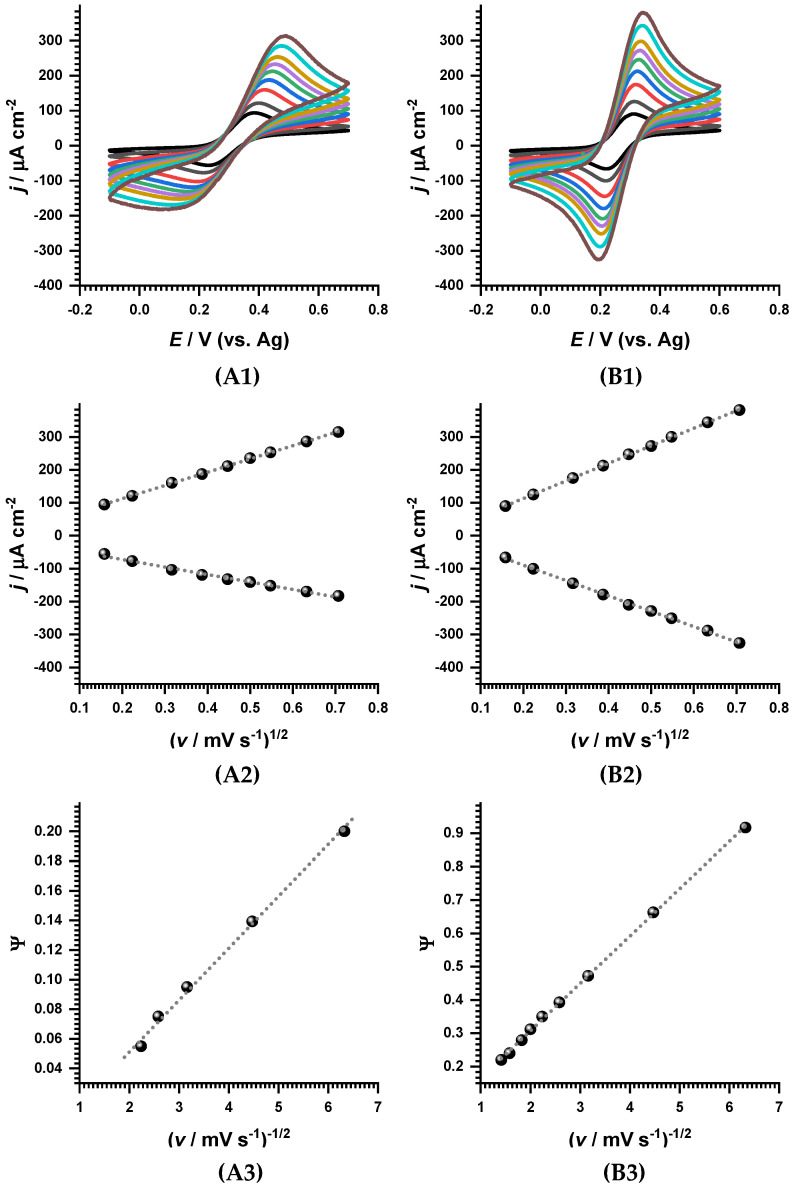
(**1**) Cyclic voltammograms at increasing scan rates (the peak current increases correspondingly) recorded in 1 mM K_4_[Fe(CN)_6_] in pH = 7.0 0.1 M phosphate buffer and (**2**) variation of the anodic and the cathodic peak currents and of the (**3**) Ψ function, indicative of the degree of reversibility, with square root of scan rate for (**A**) SPE and (**B**) mSPE.

(i) Cyclic voltammetry. The effect of the scan rate on the electrochemical response of K_4_[Fe(CN)_6_] was investigated for both electrodes between 10 and 500 mV s^−1^ (Figure 4A1,B1).

The slope of the log *j* vs. log *v* was 0.42 and 0.48 (*R*^2^ = 0.999) for SPE and mSPE, respectively, close to the theoretical value of 0.50 for an ideal process under linear diffusion conditions. Consequently, the values of both cathodic and anodic peak currents depend linearly on the square root of the scan rate, as shown in Figure 4A2,B2, in agreement with diffusion-controlled processes. The absolute value of the ratio between the currents of the anodic and the cathodic peaks |*I*_pa_/*I*_pc_| was about 1.7 and 1.1 for SPE and mSPE, respectively, specific to quasi-reversible electrochemical reactions. In agreement, the difference between anodic and cathodic peak potential values Δ*E* = *E*_pa_ − *E*_pc_ was larger than the ideal value of 57/*n* mV for fast electrode kinetics and increased with the scan rate for both electrodes.

The electroactive surface areas were calculated using the Randles–Sevick equation (Equation (1)):

*I_pa_*_/*c*_ = ±0.4463 (*F*^3^/*RT*)^1/2^ *n*(α*n*)^1/2^ *A D*^1/2^ *C v*^1/2^ (1)

where *I_p_* is the peak current in *A*, *n* the number of electrons being transferred, and *α* the charge-transfer coefficient; *C* = 1 × 10^−6^ mol cm^−3^, i.e., the concentration of K_4_[Fe(CN)_6_]; *D* = 6.3 × 10^−6^ cm^2^ s^−1^, i.e., the diffusion coefficient of K_4_[Fe(CN)_6_], and *v* is the scan rate in V s^−1^.

The *α* values for both electrodes were derived from the variation of peaks potentials, with the decimal logarithm of the scan rate *dE_p_*/*d*(*log*(*v*)) = 29.6/*αn* resulting in anodic charge-transfer coefficients of 0.36 and 0.95 for SPE and mSPE, respectively. In these conditions, the electroactive surface areas were determined to be 0.88 and 0.81 cm^2^ for SPE and mSPE, respectively.

The charge-transfers rate constants, presented in Table 2, were determined using the Nicholson method for quasi-reversible reactions limited by diffusion (Equation (2)) by plotting *Ψ* vs. *v*^−1/2^ (Figure 4A3,B3) where function *Ψ* is a quantitative measurement of the degree of reversibility of the electrochemical reaction and for which a numerical estimation is available. The values of *k*_0_ were calculated to be 0.3 × 10^−4^ s^−1^ for the SPE and 1.4 × 10^−4^ s^−1^ for the mSPE.

**Figure 5:**
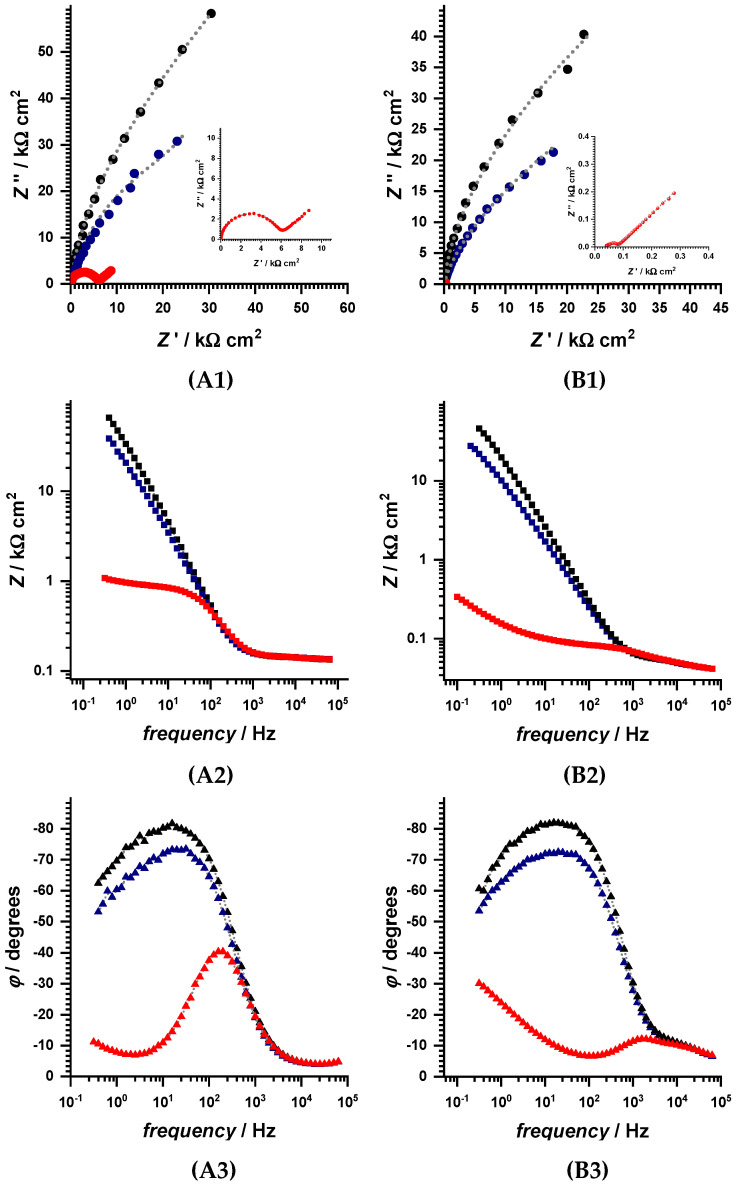
(**1**) Nyquist complex plane impedance plot and Bode representation of the variation of the (**2**) magnitude of impedance and of the (**3**) phase angle with the frequency at applied potentials of −0.10 V (black), +0.70 (blue), and +0.32 V or +0.27 V (red) for (**A**) SPE and (**B**) mSPE, respectively, in 1 mM K_4_[Fe(CN)_6_] in pH = 7.0 0.1 M phosphate buffer.

*Ψ* = *k*_0_ π^−1/2^ *D*^−1/2^ (*nF*/*RT*)^−1/2^ *v^−^*^1/2^ (2)

(ii) Electrochemical impedance spectroscopy. EIS in 1 mM K_4_[Fe(CN)_6_] in pH = 7.0 0.1 M phosphate buffer was used to investigate interfacial phenomena and predominant processes at SPE and mSPE at different applied potentials where mostly reduced or oxidized species exist at the electrode surface and at the equilibrium potential values calculated from the cyclic voltammogram at 10 mV s^−1^ where both reduced and oxidized species coexist, as shown in Figure 5 and Table 3.

**Scheme 1:**
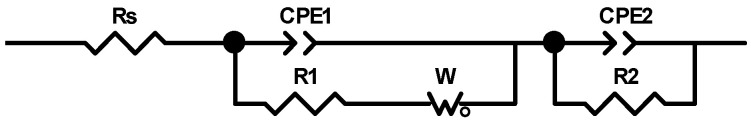
The equivalent circuit used for EIS data fitting.

Taking into consideration the electrodes’ architecture, the spectra for the SPE were fitted using equivalent circuits, which consisted of *R_s_* in series with a *R*/*CPE* combination, while for mSPE, two *R*/*CPE* were used Scheme 1.

*R_s_* represents both the ohmic drop and the resistance caused by the electrical contacts and wires. The first *R*_1_/*CPE*_1_ combination corresponds to the electrolyte/electrode interface, where *R*_1_ is correlated with the charge-transfer process at the electrolyte/electrode interface, and *CPE*_1_ is the pseudo-capacitance of the electrode in contact with solution. The second *R*_2_/*CPE*_2_ combination, which appears only in the case of mSPE, is associated with processes inside the PANI/ZnO composite, where *R*_2_ is attributed to the charge-transfer processes in the PANI/ZnO composite, while *CPE*_2_ is the constant phase element characteristic of the mixed layer. At equilibrium potential values, at medium and low frequencies, the spectra displayed a Warburg impedance *Z_w_* due to finite diffusional processes at the electrode surface, strongly influenced by the film porosity.

The results obtained after fitting the experimental data are shown in Table 3. For both electrodes, it is observed that the values of *R_s_* did not vary with the applied potential. For SPE, the resistance *R*_1_ and the capacitance of *CPE*_1_ present similar values at −0.10 and +0.70 V, meaning that no significant changes take place in the carbon layer. At +0.32 V, a lower value of the *CPE*_1_ capacitance and *R*_1_ resistance are observed due to the oxidation of Fe^2+^ to Fe^3+^. Similarly, for mSPE, resistance *R*_1_ and capacitance of *CPE_1_* present similar values at −0.10 and +0.70 V, meaning that no significant changes take place in the PANI/ZnO/carbon layer. At +0.32 V, lower values of *CPE*_1_ capacitance and *R*_1_ resistance are observed due to the redox reaction. Nonetheless, the values of *CPE*_2_ capacitance and *R*_2_ did not vary with the applied potential, showing that these fast processes that occur only at high frequency values (between 1 × 10^5^ and 5 × 10^4^ Hz) are associated with charge transfer and separation within the PANI/ZnO composite, confirming the cyclic voltammetry experiments that demonstrated that ZnO acts as a dopant of PANI, increasing its conductivity.

Comparing the electrodes, it is observed that the *R_s_* values are one order of magnitude lower in the case of mSPE. The *CPE*_1_ capacitances are similar in the absence of charge-transfer processes, but in the presence of charge-transfer reactions, the capacity of SPE is two orders of magnitude lower than in the case of mSPE, reflecting the larger charge-separation capacity of the modified electrode. The charge-transfer resistance *R*_1_ is three orders of magnitudes lower for the mSPE than for the SPE during electron-transfer reactions, which is in agreement with the faster kinetics as demonstrated by cyclic voltammetry investigations. At the same time, the diffusional resistance and diffusional time are much smaller in the case of mSPE when compared to SPE.

### 2.4. Mechanism of PANI:ZnO Conductivity

One of the main limitations of PANI application in sensing and biosensing devices is the loss of conductivity in neutral or high pH electrolytes since conductivity is highly dependent on doping with protons. However, the doping process of PANI can be also achieved by oxidizing agents.

Taking into consideration that the conduction band of ZnO is lower than the lowest unoccupied molecular orbital (LUMO) of PANI [44,45], it is proposed that ZnO acts as an oxidizing agent, and the electron transfer between PANI and ZnO leads to the formation of a *p:n* heterojunction. This was demonstrated by the cyclic voltammetry experiments where increasing the ZnO concentration while maintaining the PANI proportion with the ink led to the disappearance of the PANI redox peaks. These explanations are also confirmed by the EIS experiments, where the second *R*_2_/*CPE*_2_ combination, which appears only in the case of mSPE, is associated with processes inside the PANI/ZnO composite, where *R*_2_ is attributed to the charge-transfer processes in the PANI/ZnO composite, while *CPE*_2_ is the constant phase element characteristic of the mixed layer. Indeed, these are fast processes occurring only at high frequencies and do not vary with applied potential values.

### 2.5. Electroanalysis of Redox Amino Acids

**Figure 6:**
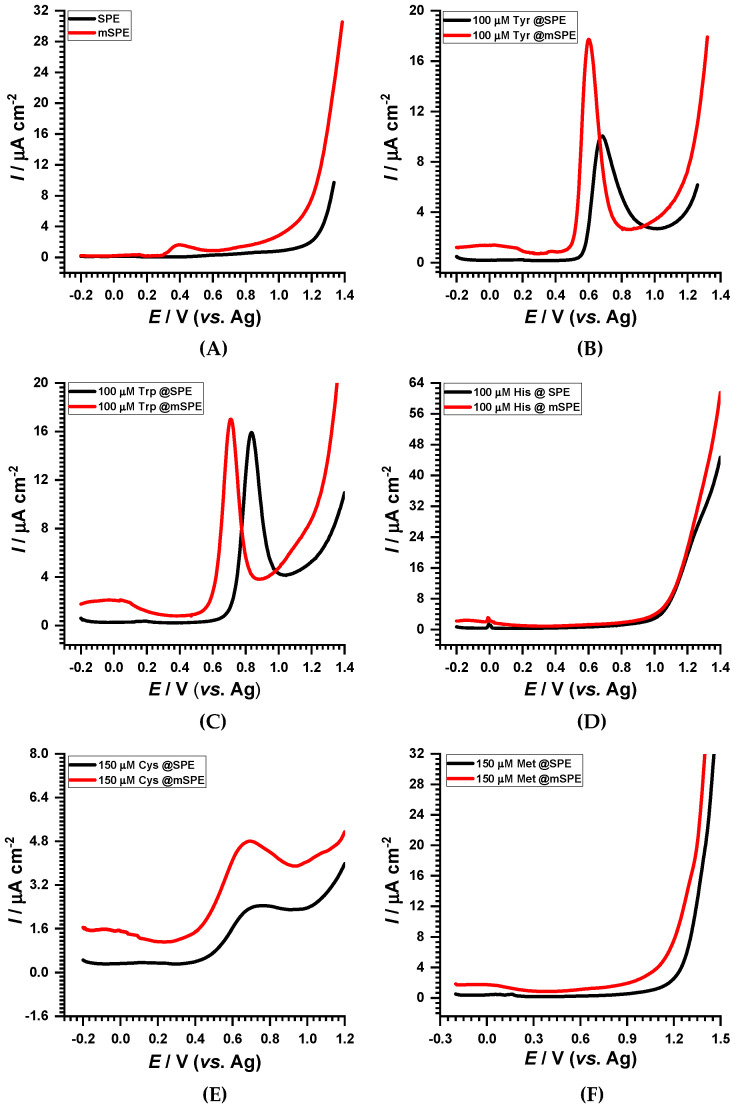
DP voltammograms obtained at SPE (▬) and mSPE (▬) in (**A**) pH = 7.0 0.1 M phosphate buffer and in solutions of 100 µM: (**B**) tyrosine, (**C**) tryptophan, (**D**) histidine, (**E**) cysteine, and (**F**) methionine.

The electrochemical behavior of all electroactive amino acids, i.e., sulfur-containing amino acids Cys and Met and the aromatic amino acids Tyr, Trp, and His, at the surface of these newly developed electrodes, SPE and mSPE, was investigated in pH = 7.0 0.1 M phosphate buffer using an amino acid concentration of 100 µM by the means of DP voltammetry (Figure 6).

Before each experiment, DP voltammograms were recorded in pH = 7.0 0.1 M phosphate buffer for SPE and mSPE. The DP voltammetry at SPE showed a profile similar to GC electrode, while for mSPE, the presence of polyaniline was observed at *E*_pa_ = +0.40 V, as shown in Figure 6A. However, in the presence of the analytes, i.e., electroactive amino acids, this peak disappeared.

The DP voltammograms recorded in 100 µM Tyr showed one oxidation peak at *E*_pa_ = +0.68 V for SPE and *E*_pa_ = +0.60 V for mSPE, whereas for 100 µM Trp, the oxidation potentials *E*_pa_ = +0.83 V at SPE and *E*_pa_ = +0.70 V at mSPE were obtained, as shown in Figure 6B,C. At both electrodes, the current/concentration (*I*/*C*) ratio showed high values (Table 4). Although the oxidation potential difference between Tyr and Trp at SPE, i.e., Δ*E*_p_ = 150 mV, was larger than the one obtained at mSPE, i.e., Δ*E*_p_ = 100 mV, the modified electrode showed a higher oxidation current for Tyr with a smaller width at the half height of the peak. The electrochemical oxidation of His at SPE and mSPE occurred at the same potential, i.e., *E*_p_ = +1.25 V, with very low oxidation currents, as shown in Figure 6D.

The DP voltammograms recorded for sulfur-containing amino acids showed that Cys and Met oxidation at *E*_pa_ = +0.70 V and *E*_pa_ = +1.27 V, respectively, occurred at the same potential for both electrodes, as shown in Figure 6E,F. Moreover, for Cys, only the first charge transfer appeared, probably due to the adsorption of the oxidation product at the electrodes surface, while for Met, only the second electrode reaction appeared, probably to the fact that at this pH, the Met oxidation peaks tend to merge [7]. At the same time, it was observed that the current/concentration (*I*/*C*) ratio was medium for Cys and low for Met for both electrodes used in this experiment, as given in Table 4.

**Figure 7:**
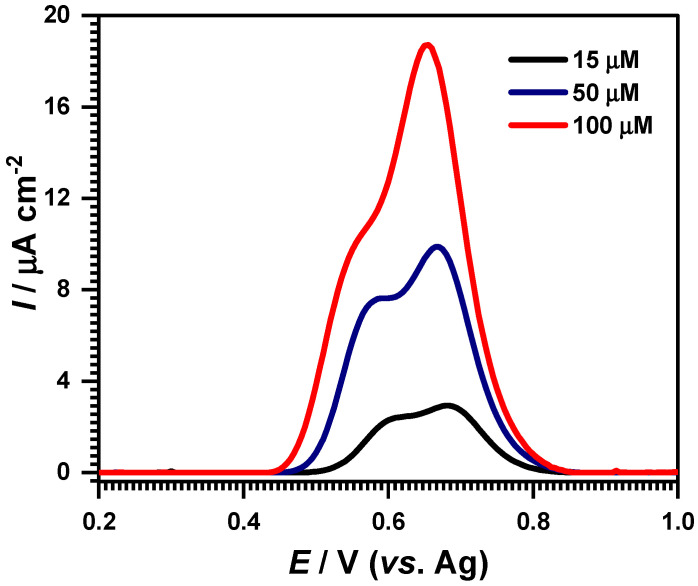
DP voltammograms obtained at mSPE for equimolar solution containing Tyr and Trp.

The DP voltammetry results presented in Figure 7 showed a good selectivity for Tyr and Trp obtained using the SPE and mSPE electrodes. Moreover, at mSPE, the oxidation reaction takes place with higher currents when compared with SPE. Therefore, in a new experiment, DP voltammograms were recorded at mSPE for equimolar solution of Tyr and Trp (Figure 7), and the separation of anodic charge-transfer reactions was obtained. The analytical parameters for the detection of Tyr and Trp using the mSPE are in Table 5. The limits of detections were calculated based on three times the standard deviation of the sensitivity, LoD = 3 × S.D.× (sensitivity)^−1^, and linearity is achieved from concentration values above 100 µM.

When compared with previous publications that involve the electrochemical detection of aromatic amino acids at electrodes modified with conductive polymers and/or ZnO nanostructures [46,47,48,49,50], it can be observed that similar limits or detection are achieved. Nonetheless, considering that the plasma levels of both amino acids are in the range of 10 to 100 µM [51,52,53], it can be concluded that the mSPE (2% PANI and 0.04% ZnO nanowires) can be successfully utilized for the simultaneous detection of Tyr and Trp in real media.

## 3. Materials and Methods

### 3.1. Materials

The electroactive amino acids tyrosine (Cat. Nr. T8566), tryptophan (Cat. Nr. T2610000), cysteine (Cat. Nr. 30089), methionine (Cat. Nr. 1.05707), and histidine (Cat. Nr. H0750000) were purchased from Merck (Darmstadt, Germany) and were used without further purification. Dimethylformamide (DMF) and PANI (polyaniline-emeraldine salt) were purchased from Merck. ZnO nanowires A90 were purchased from Novarials (Woburn, MA, USA). Carbon (ref. SCRE001), Silver (ref SCAG002), and dielectric (ref. SCINS-001) inks were provided by Mateprincs.

All necessary solutions were prepared with analytical grade reagents and purified water from a Millipore Milli-Q system (conductivity ≤ 0.1 µS cm^−1^).

### 3.2. Instrumentation

#### 3.2.1. Fabrication of Screen-Printed Carbon Electrodes

**Scheme 2:**
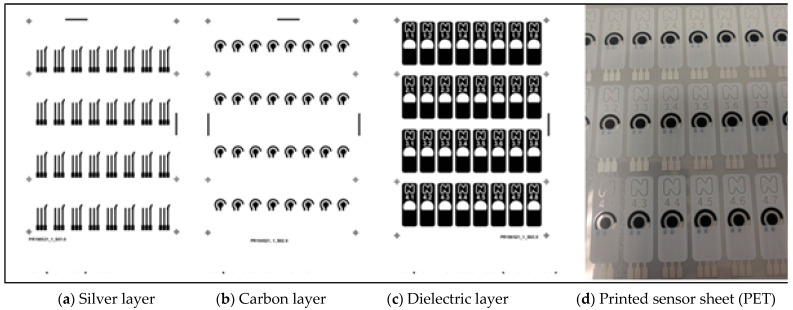
The illustration of the fabrication process of three-element electrochemical sensor. Left to right: silver layer, carbon layer, dielectric layer, and printed sensor sheet (PET).

A semi-automatic flat screen-printing machine from EKRA was used to manufacture the carbon electrodes. The prints were made on PET. The silver ink for the reference electrode was printed first. The carbon ink was printed on top as working electrode and counter electrode. Finally, a layer of dielectric ink was applied. Scheme 2 shows geometrical detail of the printed layers and final sensors. The electrodes thus obtained were characterized by cyclic voltammetry.

#### 3.2.2. Fabrication of Modified Screen-Printed Carbon Electrodes

PANI was dissolved in DMF for 24 h by mechanical stirring (Dispermat) at 500 rpm. Different percentages (*w*/*w*) by weight were tested in order to determine the most efficient composition, as presented in Table S1. Commercial ZnO nanowires were added in different amounts in order to determine the concentration that induces the higher sensitivity. TheZnO nanowires were added to PANI solution by magnetic stirring at 200 rpm for 24h. Thus, the final formula used for the modified carbon inks contained 2%(*w*/*w*) by weight PANI and 0.4% (*w*/*w*) by weight commercial ZnO nanowires. This suspension was supplied to Mateprincs for incorporation into the modified ink for use as a working electrode. For the fabrication of the electrodes, the same steps as in Section 3.2.1 were followed.

#### 3.2.3. Field-Emission Scanning Electron Microscopy (FESEM)

The morphological properties of the electrodes were investigated using a Gemini 500 Carl Zeiss (Oberkochen, Germany) field-emission scanning electron microscope (FESEM) working in both high-vacuum (HV) and variable-pressure (VP) modes from 0.2 to 30 kV, equipped with LaB_6_ filament, InLens and SE2 detectors, NanoVP mode, and a Bruker (Berlin, Germany) QUANTAX 200 energy-dispersive X-ray spectrometer (EDS) with an XFlash^®^6 silicon drift detector (SDD), an energy resolution <129 eV at Mn-Kα, and Peltier cooling.

#### 3.2.4. Raman Spectroscopy

Raman spectroscopy was performed at room temperature in a backscattering configuration using a LabRAM HR Evolution spectrometer (Horiba Jobin-Yvon, Montpellier, France) equipped with a confocal microscope and a He–Ne laser operating at 633 nm. The laser excitation power was adjusted to avoid laser-induced heating, and the laser was focused on the surface the samples using 100× objective. The calibration of the laser beam was performed on standard Si wafer by checking the Rayleigh and Raman signals.

#### 3.2.5. Voltammetric Parameters and Electrochemical Cells

Voltammetric experiments were performed using a computer-controlled Ivium potentiostat with IviumSoft version 2.219 (Ivium Technologies, Eindhoven, The Netherlands). Cyclic voltammograms were recorded using a scan rate of 100 mVs^−1^ with a 2 mV step potential, and the experimental conditions for differential pulse voltammetry (DPV) were pulse amplitude 50 mV and pulse width 100 ms at a scan rate of 5 mV s^−1^.

Electrochemical impedance spectroscopy (EIS) was performed at different applied potentials, with a perturbation of 10 mV, over the frequency range 100 kHz–0.1 Hz, with 10 frequency values per decade, and the experimental data were analyzed using the ZView software version 3.2c (Scribner Associates, Huntington Beach, CA, USA).

The equivalent circuits used for fitting the EIS data are presented in Scheme 1. *R*_s_ represents the ohmic drop and resistance caused by the electrical contacts and wires, and *R*_1_ is correlated with the charge-transfer process. The constant phase element (*CPE*) with *Z*_CPE_ = (*Ciω*)^−α^ is modelled as a non-ideal capacitor, where the capacitance *C* describes the charge separation at the double-layer interface, and the α exponent is due to the heterogeneity of the surface. The definition of the Warburg element used is *Z*_W_ = *R*_w_ (*iτω*)^−α^ cth[(*iτω*)^α^], where *R*_w_ is a diffusion resistance of electroactive species, *τ* is a time constant depending on the diffusion rate (*τ* = *l*^2^/*D*, where *l* is the effective diffusion thickness, and *D* is the effective diffusion coefficient of the specie), and α = 0.50 for a perfectly uniform flat interface.

## 4. Conclusions

The experimental study demonstrated the feasibility of a new electrochemical platform based on PANI/ZnO nanowires-modified carbon inks screen-printed electrodes as a sensitive element favoring selectivity for tyrosine and tryptophan detection. The newly developed biosensor based on PANI/ZnO nanowires-modified carbon ink was characterized by scanning electron microscopy and Raman spectroscopy in order to validate, by morphology and structure evaluations, the presence of ZnO nanowires. The electrochemical behavior was assessed by cyclic voltammetry and electrochemical impedance spectroscopy. It has been shown that the electrodes obtained from PANI/ZnO ink present better electrochemical performances than the carbon ink, such as higher charge-transfer rate constant, lower charge-transfer resistance, and higher capacitance values. The electrochemical behavior of all electroactive amino acids, i.e., sulfur-containing amino acids Cys and Met and the aromatic amino acids Tyr, Trp, and His, at the surface of new developed electrodes was investigated by means of differential pulse voltammetry. A good selectivity for Tyr and Trp was obtained at the PANI/ZnO nanowires-modified carbon ink, which allows the identification of these two amino acids in mixed solutions, an essential step forward for the electrochemical analysis of proteins.

## Figures and Tables

**Table 1:** Oxidation peak potentials and current/concentration (*I*/*C*) ratio of electroactive amino acids at glassy carbon electrode at pH 7.00 [5,6,7,8,9].

Amino Acid	Oxidation Peak Potentials (V vs. Ag/AgCl) and *I*/*C* (nA cm^−2^ µM^−1^)					
	*E*_p1_	*I*_p1_/*C*	*E*_p2_	*I*_p2_/*C*	*E*_p3_	*I*_p3_/*C*
Tyrosine	0.65	188	-	-	-	-
Tryptophan	0.65	198	1.00	47	-	-
Histidine	1.25	76	-	-	-	-
Cysteine	0.50	16	0.90	10	1.3	35
Methionine	1.05	8			-	-

**Table 2:** Electroactive area, values of slopes from plots of *j* vs. *v*^1/2^ (*b*_a_ for anodic and *b*_c_ for cathodic processes), variation of the anodic peak potential with decimal logarithm of scan rate, and the electron-transfer rate constants.

Electrode	*b*_a_ × 10^4^/ A cm^−2^(V s^−1^)^−1/2^	*b_c_* × 10^3^/ A cm^−2^ (V s^−1^)^−1/2^	Electroactive Area/ cm^−2^	*dE_pa_*/*d*(*log*(*v*/ mV s^−1^)/ mV Decade^−1^	*k*_0_ × 10^4^/ cm s^−1^
SCPe	4.02	−2.3	0.88	89.9	0.3
mSCPe	5.33	−4.66	0.81	31.5	1.4

**Table 3:** Values of equivalent circuit elements obtained by fitting the spectra in Figure 5.

	SPE			mSPE		
*E*_app_/V	−0.10	+0.32	+0.70	−0.10	+0.27	+0.70
*R*_s_/Ω cm^2^	137	145	144	39.6	36.6	39.5
*C*_1_/μF cm^−2^ s*^α^*^−1^	5.1	0.72	8.6	7.7	5.8	17.2
*α*_1_	0.92	0.91	0.86	0.94	0.80	0.88
*R*_1_/Ω cm^2^	167 × 10^3^	4.4 × 10^3^	75 × 10^3^	74 × 10^3^	26.6	34 × 10^3^
*Z*_w_/Ω s^1/2^ cm^2^	-	168	-	-	3.01	-
*τ*_W_/ms	-	2	-	-	0.1	-
*α*_W_	-	0.23	-	-	0.25	-
*C*_2_/ μF cm^−2^ s*^α^*^−1^	-	-	-	2.8	5.8	5.4
*α*_2_	-	-	-	0.87	0.92	0.82
*R*_2_/Ω cm^2^	-	-	-	14.4	14.4	15.4

**Table 4:** Oxidation peak potentials and current/concentration (*I*/*C*) ratio of electroactive amino acids at SPE and mSPE at pH 7.00.

Amino Acid	Oxidation Peak Potentials (V vs. Ag) and *I*/C (nA cm^−2^ µM^−1^)			
	SPE		mSPE	
	*E*_pa_	*I*_pa_/*C*	*E*_pa_	*I*_pa_/*C*
Tyrosine	0.68	82	0.60	166
Tryptophan	0.83	147	0.70	145
Histidine	1.25	<0.01	1.25	<0.01
Cysteine	0.70	0.03	0.70	0.05
Methionine	1.30	<0.01	1.27	<0.01

**Table 5:** Analytical parameters for the detection of Tyr and Trp in mixed solutions using the mSPE (2% PANI and 0.04% ZnO nanowires).

Amino Acid	*Sensitivity*/ µA cm^−2^ µM^−1^	*LOD*/ µM
Tyr	0.11 (S.D. = 0.234 µA cm^−2^)	6.4
Trp	0.19 (S.D. = 0.348 µA cm^−2^)	5.5

## Data Availability

Not applicable.

## Supplementary Materials

The following supporting information can be downloaded at: https://www.mdpi.com/article/10.3390/ijms24021129/s1.
