# Supplementary material for: Carbon Inks-Based Screen-Printed Electrodes for Qualitative Analysis of Amino Acids
Source: Int J Mol Sci. 2023 Jan 6;24(2):1129. doi: 10.3390/ijms24021129 (PMC9864027; doi:10.3390/ijms24021129)
Supplement: Supplementary file 1 [file ijms-24-01129-s001.zip › ijms-2014374-supplementary.pdf]

Supplementary information

Carbon inks based screen-printed electrodes for qualitative analysis of amino acids

Teodor Adrian Enache [1], Monica Enculescu [1,*], Mihaela-Cristina Bunea [1], Estibaliz Armendariz Zubillaga [2], Edurne Tellechea [2], Maite Aresti [2], María Lasheras [2], Aaron C. Asensio [3] and Victor C. Diculescu [1,*]

[1] Laboratory of Functional Nanostructures, National Institute of Materials Physics, Atomistilor 405A, 077125, Magurele, Romania
[2] NAITEC, C/ Tajonar 20 -31006, Pamplona, Navarra, Spain
[3] MATEPRINCS, Paseo Zarapuz, 1, 31200, Navarra, Spain
* Correspondence: mdatcu@infim.ro (M.E.); victor.diculescu@infim.ro (V.C.D.)

Sample electrodes with different PANI:ZnO ratios, **Table S1**, were analyzed before selecting the one presented in the manuscript (2% PANI: 0.4% ZnO).

Table S1. mSPE electrodes with different PANI:ZnO proportions.

sample #	PANI (%)	ZnO (%)
1	0	0.0
2	1	0.1
3	1	0.2
4	2	0.2
5	2	0.4
6	10	2.0

The analysis/screening of their response was performed by cyclic voltammetry in different electrolytes such as 0.1 M H_2SO_4, 0.1 M HCl, pH = 7.0 0.1 M phosphate buffer, in the absence and in the presence of 1 mM $K_4[Fe(CN)_6]$, **Figure S1**.

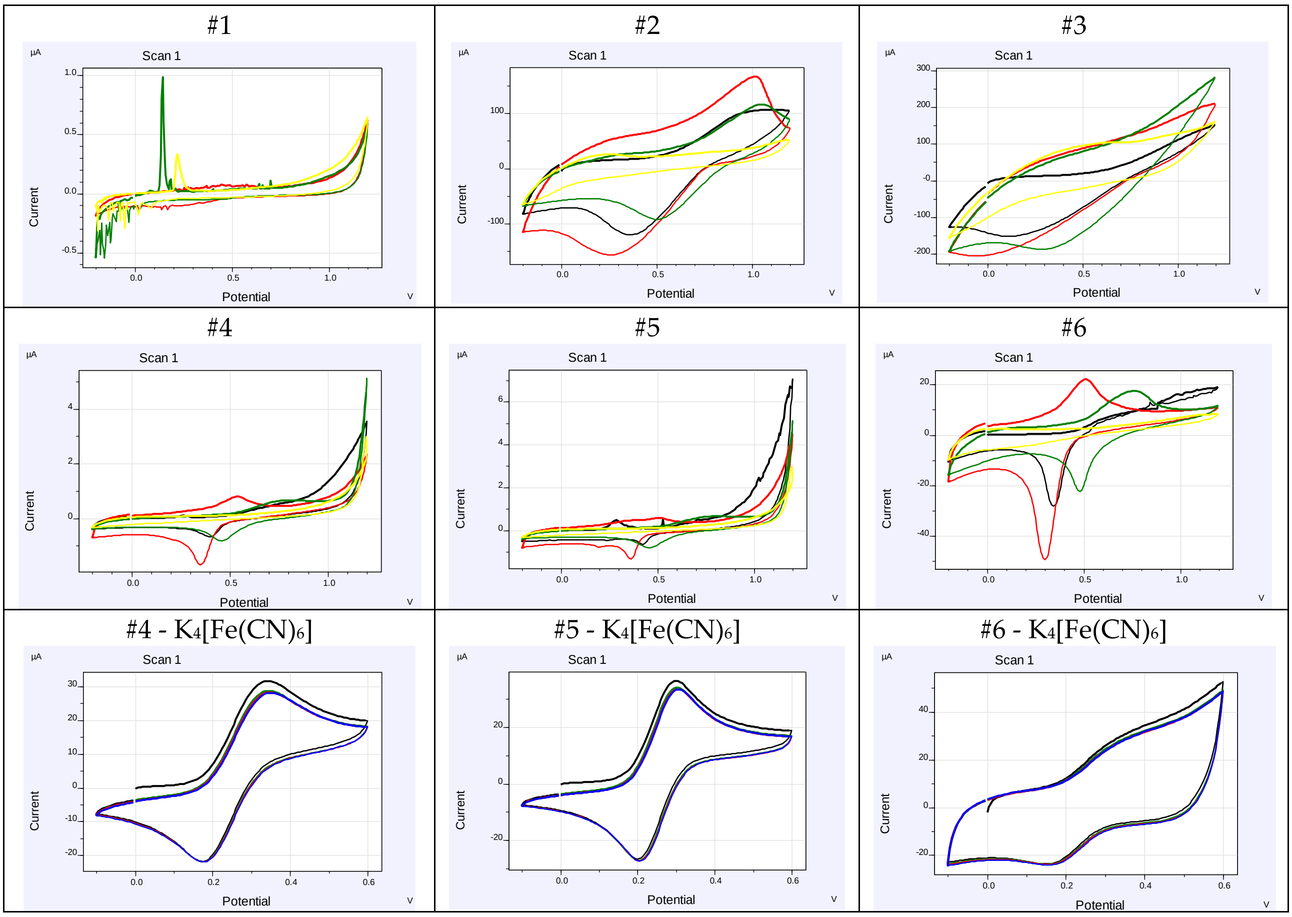

Figure S1. Cyclic voltammograms at v = 100 mV s^{-1} of different samples (Table S1) in0.1 M H_2SO_4 (red curve-first scan and black curve-the second scan), 0.1 HCl (green curve) and pH = 7.0 0.1 M phosphate buffer (yellow curve) in the absence and presence of 1 mM $K_4[Fe(CN)_6]$.

Of all these samples, the electrode with the 2% PANI : 0.4% ZnO composition has shown the most appropriate electrochemical response and chosen for further investigations.

The electrochemical behavior of SPEs and mSPE (2% PANI : 0.4% ZnO) was investigated in 0.1 M H_2SO_4 (without any redox probe) in order to assess the intrinsic electroactivity of the electrodes, **Figure S2**.

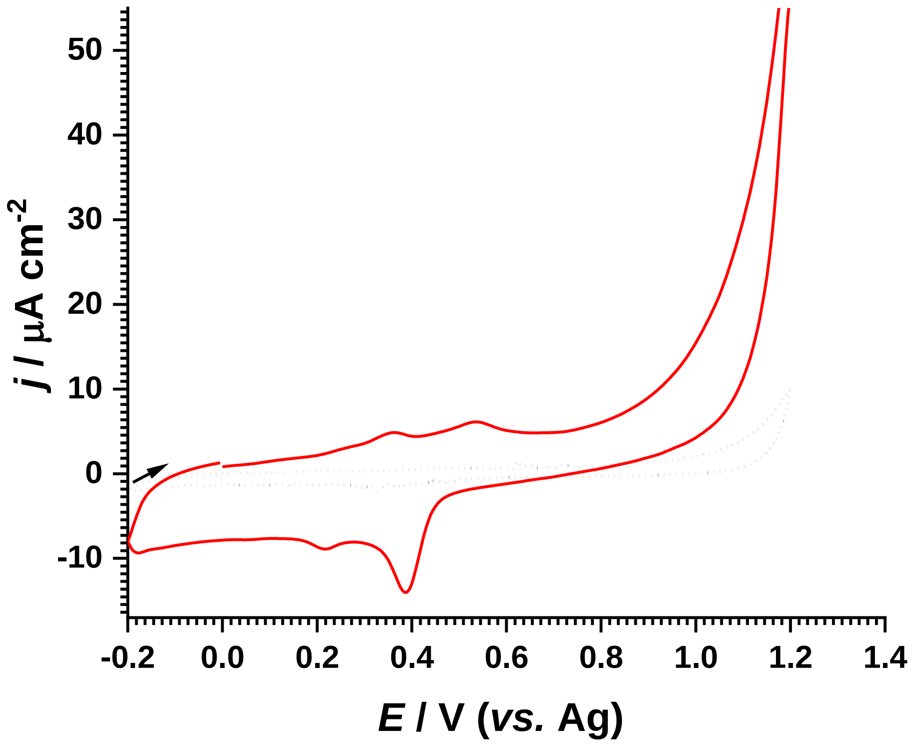

Figure S2. Cyclic voltammograms of SPE (dotted curve) and mSPE (red curve) in 0.1 M H_2SO_4 at v = 100 mV s^{-1}.
